# Integration of Primary Murine Osteocytes for In Vitro Mechanobiology Studies

**DOI:** 10.1002/cpz1.70217

**Published:** 2025-10-08

**Authors:** Kimberly Seaman, Chun‐Yu Lin, Xin Song, Amel Sassi, William W. Du, Yu Sun, Burton Yang, Lidan You

**Affiliations:** ^1^ Department of Mechanical and Industrial Engineering University of Toronto Toronto Canada; ^2^ Institute of Biomedical Engineering University of Toronto Toronto Canada; ^3^ Sunnybrook Research Institute and Department of Laboratory Medicine and Pathobiology University of Toronto Toronto Canada; ^4^ Department of Mechanical and Materials Engineering Queen's University Kingston Canada

**Keywords:** bone metastasis, fluid flow, mechanobiology, microfluidics, osteocytes, primary cells

## Abstract

This article describes the isolation, culture, and integration of primary murine osteocytes on a microfluidic platform for in vitro mechanobiology studies. Osteocytes are mechanosensitive cells embedded within the bone matrix and regulate bone remodeling. The MLO‐Y4 osteocyte‐like cell line typically used for mechanobiology studies can provide a good physiological response to mechanical stimulation but has been shown to have missing or low levels of key osteocyte markers found in vivo, such as sclerostin. However, primary osteocytes are difficult to isolate and study in vitro due to their location within the bone matrix, and the scope of existing protocols for osteocyte extraction only includes the isolation of primary cells using collagenase. Here, optimized protocols for the isolation, culture, and real‐time calcium imaging of primary osteocytes in response to oscillatory fluid flow are outlined. Moreover, we discuss how to incorporate and maintain primary osteocytes on a physiologically relevant microfluidic platform for in vitro bone metastasis studies. In calcium imaging and microfluidic experiments, primary osteocytes are compared to the MLO‐Y4 cell line as a control or reference group. Primary osteocytes can be extracted in 6 to 8 hr and typically require 1 week to recover before use in experiments. Calcium imaging of primary osteocytes can be completed in 24 hr. The time required for microfluidic culture varies on the platform used; the microfluidic experiment described here takes 6 days to complete. We anticipate that the information from this protocol will aid other researchers with incorporating primary osteocytes to enhance the biological relevance of in vitro studies on bone mechanobiology and disease. © 2025 The Author(s). Current Protocols published by Wiley Periodicals LLC.

**Basic Protocol 1**: Isolation of primary murine osteocytes using Liberase TM

**Basic Protocol 2**: Primary osteocyte subculture and E11/podoplanin staining

**Basic Protocol 3**: Real‐time calcium imaging of primary osteocytes

**Basic Protocol 4**: Integration of primary murine osteocytes onto a microfluidic platform for observing bone metastasis

## INTRODUCTION

Osteocytes are terminally differentiated cells that comprise 95% of all cells found in bone tissue (Schaffler & Kennedy, 2012). Osteocytes are in the lacunocanalicular network within the bone matrix and are highly sensitive to mechanical stimuli (Schaffler & Kennedy, 2012). When subjected to mechanical loading through movement or exercise, osteocytes sense interstitial fluid flow generated in the lacunocanalicular system and transduce these signals into biochemical factors that regulate bone effector cell activities (osteoblasts and osteoclasts) (Bonewald, 2011; Fritton & Weinbaum, 2009). This process is known as mechanotransduction, which involves a series of signaling cascades in osteocytes. Osteocytes possess several cellular detection mechanisms that allow them to sense fluid shear stress, including integrins, the pericellular matrix, primary cilia, ion channels, and gap junctions or hemichannels (Qin et al., 2020). Initial mechanotransduction signal transmission includes an influx of calcium ions, and production of adenosine triphosphate and nitric oxide (Li et al., 2021; Uda et al., 2017). Osteocyte effector response in the later stages of signal transmission includes the release of prostaglandin E2 and osteoprotegerin, and reduced expression of receptor activator of nuclear factor kappa‐B ligand, and Wnt signaling inhibitors sclerostin and dickkopf‐1 (Jacobs et al., 1998; Klein‐Nulend et al., 1995; You et al., 2008).

In vitro studies of osteocytes are critical to understanding bone remodeling, mechanotransduction and the pathology of bone diseases, such as osteoporosis or bone metastases. However, osteocytes are difficult to isolate and study in vitro because of their terminal status and location within the bone matrix (Zhang et al., 2019). The establishment of osteocyte cell lines such as MLO‐Y4 has enabled mechanobiology studies in vitro, although this cell line lacks the expression of key osteocyte markers found in vivo such as sclerostin (Kato et al., 1997). While the OCY454 osteocyte cell line expresses appreciable quantities of sclerostin, use of these cells for in vitro mechanobiology studies is limited by its inconsistent calcium response under physiological flow (Xu et al., 2019). Moreover, OCY454 cells require culture at a permissive temperature (33°C) to enable proliferation and a 10‐day differentiation period at a semi‐permissive temperature (37°C), thereby prolonging the time taken to perform in vitro experiments (Xu et al., 2019). Therefore, use of primary osteocytes can serve as a method to enhance the biological relevance of in vitro osteocyte mechanobiology studies. However, the isolation and use of primary osteocytes can be challenging, and the scope of existing extraction protocols only includes the isolation of primary osteocytes (Gooi et al., 2019; Shah et al., 2016; Stern et al., 2012). Herein, we provide an optimized osteocyte isolation protocol from our laboratory as a potential alternative to the use of collagenase type I enzyme, as well as protocols for the subculture (for seeding experiments) and real‐time calcium imaging of primary osteocytes. Additionally, we discuss the incorporation and maintenance of primary osteocytes in a microfluidic platform for studying early‐stage bone metastasis as demonstrated in the study by Seaman et al. (2025). Ultimately, we anticipate this protocol will provide a more comprehensive overview of the isolation, culture, and use of primary osteocytes to enhance the biological relevance of in vitro mechanobiology studies.

**Figure 1:**
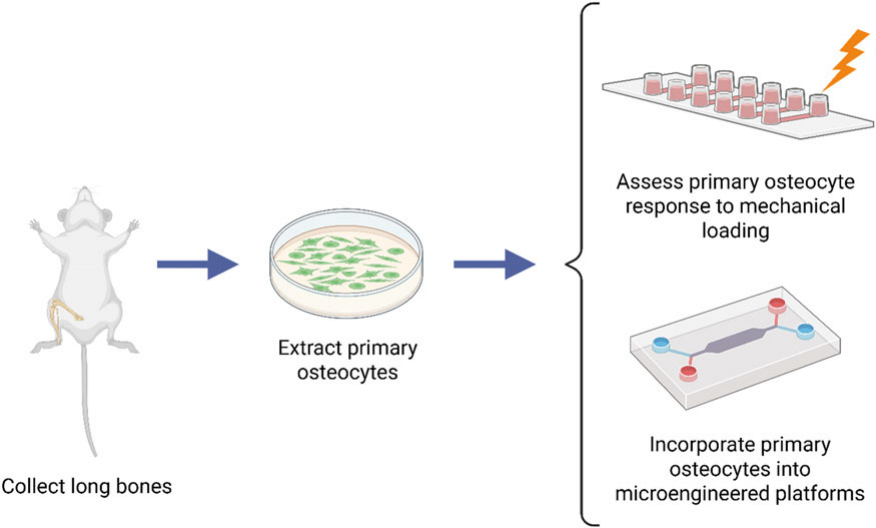
Workflow for integrating primary osteocytes for in vitro bone mechanobiology studies. Here, we describe experiments involving primary osteocytes that involve assessing their response to mechanical loading and/or their incorporation into physiologically relevant microengineered platforms to study various pathologies such as bone metastases.

*NOTE*: Studies involving animals must be reviewed and approved by the relevant institutional Animal Care and Ethics Committee.

*NOTE*: All animals were handled in accordance with the relevant guidelines approved by the Animal Care Committee of Sunnybrook Research Institute (AUP #2022‐224).

Basic Protocol 1 describes the extraction of primary osteocytes from murine long bones. Basic Protocol 2 describes the subculture procedure for primary osteocytes for seeding experiments as well as the immunofluorescence staining procedure for the osteocyte‐specific marker E11/podoplanin. Basic Protocol 3 describes the real‐time calcium imaging of primary osteocytes to confirm these cells could still produce a functional response to mechanical loading post‐extraction. Basic Protocol 4 outlines the protocol for establishing microfluidic experiments to study cancer cell extravasation towards bone and incorporation of primary osteocytes onto this microfluidic platform. A high‐level experimental workflow for these protocols is presented in Figure 1.

## Basic Protocol 1 ISOLATION OF PRIMARY MURINE OSTEOCYTES USING Liberase TM

This protocol describes the extraction of primary murine osteocytes from long bones upon collection of tissue samples. The protocol provides a step‐by‐step explanation of the processing and preparation of bone samples, followed by a series of digestions using Liberase TM (a high purity blend of collagenases) and EDTA. Completion of this protocol should result in the extraction of primary osteocytes that can eventually be used for experiments in ∼1 week. Previously published protocols on primary osteocyte extraction from mice exist (Gooi et al., 2019; Shah et al., 2016; Stern et al., 2012). Here, we detail the steps that were taken by our research group to extract primary cells and obtain the results shown in Seaman et al. (2025), as different enzymes and reagents were used for this protocol.

### Materials

Primary osteocyte medium (see recipe)
DPBS/BSA solution for transport (see recipe)
Mice, C57BL/6, 2‐ to 24‐months‐old, male or female
Hank's balanced salt solution (HBSS) (Gibco, 14025092)
α‐MEM + 10% penicillin‐streptomycin (P/S) sterilizing solution (see recipe)
Liberase TM solution (see recipe)
1× Dulbecco's phosphate buffered saline (DPBS) (Sigma‐Aldrich, D8537‐500ML)
EDTA solution (see recipe)
Collagen‐coated plates (see recipe)

100‐mm culture dishes (Corning, 430167)
50‐ml conical tubes (sterile) (Sarstedt, 62.547.205)
Gauze (sterile, individually packaged)
Autoclaved surgical instruments, i.e., tweezers, scalpel, scissors, forceps
Container with ice
Biosafety cabinet (Thermo Scientific, Model 1355 Class A2)
6‐well plates (Sarstedt, 83.3920)
1‐ml syringe, sterile, Luer‐Lok tip (BD, 309628)
25‐ or 27‐gauge needle (BD, 305122 or 305109, respectively)
100‐ml glass beaker
Cell culture incubator, 37°C and 5% CO_2_ (Thermo Scientific Forma Steri‐Cycle CO_2_ Incubator, 184 L)
Orbital shaker (Labline, 3520)
P1000 pipette
15‐ml tube (sterile) (Sarstedt, 62.554.205)
Centrifuge (Eppendorf, Centrifuge 5810)

1 Fill two Petri dishes with ∼20 ml primary osteocyte medium each and fill 50‐ml tubes (1 tube per 1 to 2 mice) with ∼15 ml DPBS/BSA solution for transport (or more as needed to submerge all samples in the tube). Place a piece of sterile gauze to soak in medium in one of the two Petri dishes.
2 Obtain long bones from freshly sacrificed mice.
  a. Femurs and tibiae can be separated at the joint; however, each bone should ideally remain intact.
  b. Place the long bones in the 100‐mm Petri dish with medium.
  c. Cut the tendons as well as the bulk of the muscle off the bones using a scalpel.
  d. Remove as much soft connective tissue (tendons, ligaments) as possible using a scalpel or surgical scissors.
  e. Gently peel the muscle using the gauze soaked in primary osteocyte medium to help the removal of excess tissues. Do not do this for >5 min.
  f. Once cleaned, place the bone samples in the corresponding 50‐ml tube with DPBS/BSA on ice.
    Steps 1 and 2 can be performed on a lab bench. We highly recommend preparing at least two sets of autoclaved surgical instruments: one set for the lab bench and another set for the sterile laminar flow hood. Ensure that bone samples are always submerged in liquid (either primary osteocyte medium or DPBS/BSA solution). Bone samples should never be left to dry or be exposed to air for excessive periods of time. Note that if samples need to be transported longer distances to the laminar flow hood, we recommend leaving muscle on the long bone samples for transport (still submerged in a DPBS/BSA solution on ice), then cleaning just prior to prepping for digestions. The Petri dish with primary osteocyte medium and gauze can be refreshed after processing 2 to 3 mice or as needed.
3 In a sterile biosafety cabinet:
  a. Place 3 ml HBSS per well in 6‐well plates.
  b. The number of wells needed depends on the number of samples; for this step, there should be two wells filled with HBSS per mouse (or sample).
  c. Place the bone samples in a well with HBSS and finish cleaning off any remaining soft tissue on the long bone samples using surgical scissors and a scalpel.
  d. Scrape off the periosteum of each bone using a scalpel.
  e. Place the samples in another fresh well with HBSS to rinse off the remaining excess soft tissue.
    We recommend determining the number of wells required and labeling the wells of the 6‐well plate with the sample number or label beforehand to keep track of samples in a time‐efficient manner for Steps 3 to 6. This is especially important if mice of different ages/strains/sex are being use for experiments, and because samples should be processed for digestion as quickly as possible to enhance the yield of viable cells.
4 Fill as many 6‐well plates with 3 ml HBSS as needed.
  a. In the fresh wells from step 3, remove the epiphyses with surgical scissors and flush out the bone marrow with HBSS using a 1‐ml Luer‐Lok syringe connected to a 25‐ or 27‐gauge needle.
  b. Flush out the marrow into a glass beaker until the liquid (HBSS) runs clear and the red marrow is visibly absent from the bone.
  c. Place the cleaned bone into another fresh well with HBSS.
5 Perform the following:
  a. Prepare a 6‐well plate with 4 ml α‐MEM + 10% P/S sterilizing solution added per well for each sample.
  b. Prepare a 6‐well plate with 3 ml HBSS per well as needed.
  c. Place the bone samples in the wells with the α‐MEM + 10% P/S solution for 1 min using tweezers to sterilize the samples.
  d. Gently shake the well plate during the 1‐min incubation.
  e. Promptly remove the samples with a fresh pair of tweezers and place into wells containing 3 ml fresh HBSS.
6 Prepare a fresh 6‐well plate for digestion by adding 2 ml HBSS into each well as needed.
  a. Transfer the long bones of 1 to 2 mice into one well with a pair of tweezers.
  b. Cut the bone samples into 1‐ to 2‐mm pieces using a pair of surgical scissors and forceps in the HBSS.
  c. Once all the bones are cut, add 2 Wünsch units of Liberase TM into each well.
    Note that while long bones from multiple mice can be pooled together for cleaning or processing, each well with Liberase should only contain the long bones from 1 to 2 mice for optimal enzymatic digestion.
7 Incubate the bone fragments in the 6‐well plate at 37°C and 5% CO_2_ with shaking at 200 RPM for 20 min. This is the first digestion, known as Fraction 1.
  While optional, we highly recommend quickly taking a brightfield picture of one of the wells with cells after each digestion. We do this to keep track of the number of digestions that have been performed so far.
8 Remove the supernatant from Fraction 1 and wash the bone fragments with 2 ml HBSS three times.
  To remove liquid from the wells, it is recommended to use a P1000 pipette rather than an aspirator so as not to aspirate any bone fragments.
9 After the third wash, add 2 ml HBSS and 2 Wünsch units of Liberase TM to each well. Incubate for 20 min again as described in step 7.
10 Remove the supernatant from Fraction 2 and wash each well three times with 2 ml DPBS (–Mg and –Ca).
  Note that HBSS is used in washes when the subsequent digestion involves Liberase TM, and that DPBS –/– is used in washes where the subsequent digestion involves EDTA. This is because EDTA removes Ca from bone fragments; having Ca in the buffer is detrimental to the function of EDTA in removing Ca from bone. On the other hand, Mg and Ca are required in the buffer for optimal enzymatic (Liberase TM) activity; thus, HBSS with Ca and Mg is used as a buffer with Liberase TM. If you suspect that three washes may not be removing Ca sufficiently, an additional wash can be added.
11 Add 2 ml EDTA solution and incubate at 37°C and 5% CO_2_ with shaking at 200 RPM for 30 min.
  Note the difference in incubation time using Liberase TM (20 min) and EDTA solution (30 min).
12 Remove the supernatant from Fraction 3 and wash the fragments in each well with 2 ml HBSS three times.
13 Add 2 ml HBSS with 2 Wünsch units of Liberase TM and incubate for 20 min as described above.
14 Remove the supernatant from Fraction 4 and wash the fragments in each well with 2 ml DPBS three times.
15 Add 2 ml EDTA solution and incubate for 30 min as described above.
16 Remove the supernatant from Fraction 5 and wash the fragments in each well with 2 ml HBSS three times.
17 Add 2 ml HBSS with 2 Wünsch units of Liberase TM and incubate for 20 min as described above.
18 Remove the supernatant from Fraction 6 and wash the fragments in each well with 2 ml HBSS three times.
19 Add 2 ml HBSS with 2 Wünsch units of Liberase TM and incubate for 20 min as described above.
20 Collect the supernatant from Fraction 7 from each well into a 15‐ml tube (use one 15‐ml tube per well). Wash the fragments with 2 ml DPBS three times. Collect the liquid from each of the washes into the 15‐ml tube. The total volume of liquid in each 15‐ml tube should be ∼8 ml.
21 Spin down the 15‐ml tubes in a centrifuge for 10 min at 200 × *g*, room temperature, to pellet cells. Gently aspirate the supernatant and replace with fresh primary osteocyte medium. Resuspend the cell pellet and plate the cells in a collagen‐coated 100‐mm dish filled with primary osteocyte medium.
  We recommend placing the dishes in the cell culture incubator beforehand to allow the medium to equilibrate to 37°C and the optimal pH. Note that each cell culture dish will contain the cells from Fractions 7 to 9, therefore, dishes should be labeled accordingly such that primary cells are plated from the same well.
22 Add 2 ml EDTA solution and incubate bone fragments for 30 min as described above.
23 Collect the supernatant from Fraction 8 into a 15‐ml tube and wash the fragments with 2 ml HBSS three times. Collect the liquid from the washes again in the same 15‐ml tubes. Spin down the cells and plate into the collagen‐coated 100‐mm dishes using the same settings as described in step 21.
24 Add 2 ml HBSS with 2 Wünsch units of Liberase TM and incubate for 20 min as described above.
25 Collect the supernatant from Fraction 9 into a 15‐ml tube and wash the fragments with 2 ml HBSS three times. Collect the liquid from the washes again in the same 15‐ml tubes. Spin down and plate cells into the corresponding collagen‐coated 100‐mm dish.
26 Culture the plated primary cells from Fractions 7 to 9 at 37°C and 5% CO_2_.
  a. Avoid disturbing the plates to allow cells to attach during the first 48 hr after extraction.
  b. If cells are still not attaching after 2 days, add 2 to 3 ml of primary osteocyte medium with a P1000 pipette using the wall of the culture dish to minimize disturbing cells any further.
  c. Perform a full or partial medium change (if cells are still attaching) after 4 days.
    The residues from the extraction reagents (Liberase TM enzymes and EDTA) are harmful to cells and can affect the yield of viable primary cells. These reagents can be diluted during the first few days after extraction by gently adding a few ml of primary osteocyte medium into the dishes in the first days following extraction. Do not completely remove the medium until cells have attached to the plate; if cells are still attaching after 4 days, a partial medium change can be performed. We recommend using a P1000 pipette for medium changes to avoid disturbing cells.

## Basic Protocol 2 PRIMARY OSTEOCYTE SUBCULTURE AND E11/PODOPLANIN STAINING

This protocol describes the subculture procedure of primary osteocytes for experiments as well as the staining protocol for E11/podoplanin, an osteocyte‐specific marker to determine the population of osteocytes within the primary cell cultures. Following the protocol allows for a high yield of passaged primary cells for the seeding of in vitro experiments as well as the validation of osteocyte‐specific markers. We share this protocol to provide further details on subculturing primary osteocytes, as we initially encountered challenges with detaching cells from culture plates for experiments that resulted in lower cell yields than anticipated.

### Materials

Isolated primary osteocytes cultured in 100‐mm dishes (see Basic Protocol 1)
DPBS (Sigma‐Aldrich, D8537‐500ML)
Trypsin‐EDTA (Gibco, 2520072)
Primary osteocyte medium (see recipe)
Trypan blue (Gibco, 15250061)
Collagen‐coated plate (100‐mm dish or a well‐plate) (see recipe)
4% methanol‐free paraformaldehyde (Thermo Scientific, 28908)
0.1% Triton X‐100 (Sigma‐Aldrich, T8787‐50ML)
Blocking buffer (immunofluorescence) (see recipe)
E11/podoplanin primary antibody solution (see recipe)
Alexa Fluor 488 secondary antibody solution (see recipe)
4',6‐diamidino‐2‐phenylindole (DAPI) (Cell Signaling Technology, 4083)

Biosafety cabinet (Thermo Scientific, Model 1355 Class A2)
Aspirator (Gast, 0523‐101Q‐G180DX)
Cell culture incubator, 37°C and 5% CO_2_ (Thermo Scientific Forma Steri‐Cycle CO_2_ Incubator, 184 L)
Tissue culture microscope (Zeiss, Z‐AXIO40C)
Serological pipettes
15‐ml conical tube (Sarstedt, 62.554.205)
Hemacytometer (Sigma‐Aldrich, Z359629)
Centrifuge (Eppendorf, Centrifuge 5810)
48‐well plate (Corning, 3548)
Fluorescent microscope for imaging (Nikon Eclipse Ti‐U inverted microscope)

1 In a biosafety cabinet, aspirate medium from the 100‐mm cell culture dish.
2 Gently wash dishes with 10 ml warm DPBS for 20 s and aspirate the supernatant.
3 Add 4 ml Trypsin‐EDTA to the cell culture dish. Incubate at 37°C at 5% CO_2_ for 5 min. Do not disturb the samples or open the incubator for optimal cell detachment.
4 Check under the cell microscope to see if cells have detached. Most cells should be detached from the plate or can be detached mechanically by carefully tapping the sides of the dish with your hands.
  If cells have not detached, more trypsin (1 to 2 ml) may be required to detach cells over one incubation. Cultures can be re‐incubated for an additional 1 to 2 min, however, this is not ideal as cultures have already been trypsinized for 5 min.
5 Neutralize the trypsin with 4 ml primary osteocyte medium. Create a single cell suspension using a serological pipette and transfer into a 15‐ml tube.
6 Take 20 µl of the cell suspension for counting. To count cells, mix with 20 µl Trypan blue and dispense 10 µl of the mixture into a hemacytometer.
  The yield of primary cells varies; typically, we observe 1–2 × 10^6^ cells from one 100‐mm dish after extraction. Cells can be used for experiments up to 10 days after extraction and replated for subsequent seeding of other experiments during this timeframe. We do not recommend replating if there are <2.5 × 10^5^ cells remaining.
7 Spin down the cell suspension in the centrifuge for 7 min at 250 × *g*, room temperature, to pellet cells.
8 Aspirate the supernatant and resuspend cells in primary osteocyte medium to the desired concentration.
9 Proceed to step 10 for osteocyte marker confirmation, or to Basic Protocols 3 or 4 for in vitro experiments.
10 Seed cells in a collagen‐coated dish or well plate at a density of 7500 cells/cm^2^. Ensure that cells are distributed evenly.
  The volumes described in steps 12 to 20 are for a 48‐well plate (Corning, 3548). However, other dishes can be easily substituted assuming the liquid volumes used fall under recommended guidelines specified by the manufacturer (typically, 0.2 to 0.3 ml medium per square centimeter of culture surface area).
11 Culture cells overnight.
12 Wash cells in DPBS (200 µl per well). Fix samples in 4% methanol‐free paraformaldehyde (200 µl per well) for 15 min at room temperature.
13 Rinse samples in DPBS three times (200 µl per well). Incubate each rinse for 5 min to remove all excess fixative.
14 Permeabilize samples by incubating in a 0.1% Triton X‐100 solution (200 µl per well) for 10 min. Rinse samples in DPBS (200 µl per well) three times for 5 min each.
15 Incubate samples in a blocking buffer containing BSA and normal serum diluted in DPBS (200 µl per well) for 1 hr at room temperature.
16 Incubate samples with the primary E11/podoplanin antibody solution (1:100 dilution) overnight at 4°C (200 µl per well).
17 Wash samples in DPBS (200 µl per well) three times for 5 min.
18 Incubate samples in the secondary antibody solution (1:100 dilution) (200 µl per well) in the dark for 2 hr at room temperature.
19 Wash samples in DPBS (200 µl per well) three times for 5 min.
20 Counterstain samples with 1 µg/ml DAPI (200 µl per well) for 10 min in the dark. Remove DAPI stain and replace with fresh DPBS (300 µl per well).
21 Image samples.

## Basic Protocol 3 REAL‐TIME CALCIUM IMAGING OF PRIMARY OSTEOCYTES

This protocol describes the seeding and preparation of osteocyte samples for real‐time calcium imaging to assess their response to fluid shear stress following the extraction process. We utilized this assay to determine whether extracted primary osteocytes could produce a functional response to mechanical loading associated with their phenotype following the isolation process. The cytoplasm of primary cells is stained with FURA‐2 AM, a ratiometric calcium ion indicator, and oscillatory fluid flow is applied to stimulate mechanotransduction. Completion of the protocol allows for the observation of the live calcium response to flow, and the collection and analysis of data to assess the percentage of responding cells and the magnitude of the calcium response.

### Materials

0.15 mg/ml collagen solution (see recipe)
DPBS (Sigma‐Aldrich, D8537‐500ML)
Primary osteocytes (see Basic Protocol 2)
Primary osteocyte medium (see recipe)
Working medium for imaging primary osteocytes (see recipe)
Fura‐2 AM dye (abcam, ab120873)
Dimethyl sulfoxide (DMSO) (Sigma‐Aldrich, D2650‐100ML)
α‐MEM (Gibco, 12571063)

Biosafety cabinet (Thermo Scientific, Model 1355 Class A2)
Ibidi µ‐Slide VI 0.4 channels (ibidi, 80606)
Cell culture incubator, 37°C and 5% CO_2_ (Thermo Scientific Forma Steri‐Cycle CO_2_ Incubator, 184 L)
Vortex mixer (Fisher Scientific, Standard 120V)
15‐ml conical tube (Sarstedt, 62.554.205)
1‐ml syringe, sterile, Luer‐Lok tip (BD, 309628)
Polyethylene tubing, autoclaved, 0.048‐in. outer diameter (VWR, CA‐63018‐689)
Ibidi elbow connector (ibidi, 10802)
Fluorescent microscope for imaging (Nikon Eclipse Ti‐U inverted microscope)
Linear actuator, custom‐made
  A 2.3‐mm stroke length to deliver 2 Pa shear stress was used considering a 4.78‐mm inner diameter of with the BD 1‐ml Luer‐Lok tip syringe.
Ratiometric imaging software (EasyRatioPro)

1 In a biosafety cabinet, designate and label the inlet and outlet ports of ibidi µ‐Slide VI 0.4 channels.
  Note that because samples will be incubated and imaged one at a time, we recommend only seeding one channel per ibidi device. If the lid stays on the remaining channels at all times, the ibidi devices can be stored in sterile conditions and the remaining empty channels can be used in subsequent calcium imaging experiments.
2 Coat ibidi µ‐Slide VI 0.4 channels in a 0.15 mg/ml type I rat tail collagen solution for 1 hr at room temperature. Each channel requires ∼75 µl of the collagen solution.
  Avoid creating bubbles in the ibidi channels during the coating and cell seeding steps to ensure a uniform distribution of cells.
3 After 1 hr, rinse the channels with cold DPBS three times (∼100 µl per rinse).
4 Rinse the channels another three times in 100 µl primary osteocyte medium.
5 Passage primary cells as described in Basic Protocol 2 and create a single cell suspension of primary osteocytes at a density of 250,000 cells/ml.
6 Slowly add 100 µl of the cell suspension to an ibidi channel. Upon adding the cell suspension, gently remove excess medium from the outlet port. There should be little to no medium in the inlet and outlet ports.
7 Incubate the ibidi channels with the cell suspension at 37°C and 5% CO_2_ for 1 hr to allow cells to attach.
8 Prepare working medium for calcium imaging the next day (see recipe).
  If there are still cells not attached after 1 hr, samples can be incubated for an additional 15 to 30 min.
9 Add 100 µl of fresh primary osteocyte medium to each channel.
10 Culture primary cells overnight.
11 After overnight culture, prepare the FURA‐2 AM stain for calcium imaging. In dark or low light, add 50 µl DMSO to one vial of FURA‐2 AM powder.
12 Mix the solution thoroughly using a vortex mixer and pipette tip.
13 Add the concentrated dye solution (∼50 µl) to 5 ml working medium in a 15‐ml tube. Mix thoroughly.
  Note that samples for calcium imaging are prepared one a time, so steps 14 to 21 need to be repeated for every sample. The staining of samples can be staggered in a manner such that when one sample is being imaged, the next sample is being incubated with FURA‐2 AM. However, staggering sample preparation depends on the number of people performing the calcium experiment and familiarity with the calcium imaging protocol.
14 Rinse the sample to be stained with FURA‐2 AM twice in DPBS (125 µl per rinse). Gently add 90 µl of the FURA‐2 AM staining solution three times into the channel to ensure proper staining of cells.
  Always mix the FURA‐2 staining solution before staining each sample to ensure a uniform distribution of the dye components.
15 Incubate the sample with the staining solution for 35 min at room temperature.
16 Rinse the cells four times in DPBS (125 µl) and three times in working medium (100 µl) to completely remove the FURA‐2 dye.
  Note that samples must be incubated at room temperature to exclusively stain the cytoplasm for calcium imaging. Incubating samples at 37°C allows for FURA‐2 to permeate into organelles.
17 Connect tubing to a 1‐ml Luer‐Lok syringe filled with α‐MEM basal medium (no serum or P/S). Ensure there are no bubbles in the tubing.
18 Connect the tubing to the inlet of the ibidi channel. Ensure that no bubbles are formed at the connection.
  Prevent bubble by creating a small 50 µl droplet at the elbow connector, then connecting to the inlet to form a liquid–liquid interface.
19 Bring the sample to the fluorescent microscope and let cells equilibrate for 20 min on a heated microscope stage set to 33°C before imaging. During this time, connect the 1‐ml syringe to the linear actuator that is set to apply a peak shear stress of 2 Pa at a frequency of 1 Hz.
  While connecting the syringe to the linear actuator, do not move the syringe plunger as this will disturb cells during their equilibration time.
20 Determine an arbitrary area to be selected for calcium imaging. This area should be in the middle of the channel width to observe calcium response at peak shear stress. Primary osteocytes should be spread out and exhibit a dendritic morphology.
21 Begin recording the calcium response of primary cells over a total of 5 min using EasyRatioPro software. Take a static baseline reading for 1 min. Then, apply oscillatory fluid flow (2 Pa, 1 Hz) for 3 min by turning on the linear actuator. Turn off the linear actuator for the final 1 min to record the return to baseline.
22 Analyze the ratiometric data (340 nm/380 nm) to determine the calcium response of osteocytes. We consider the calcium response of primary osteocytes to be significant only if the magnitude of the response was twice as high as the static baseline.

## Basic Protocol 4 INTEGRATION OF PRIMARY MURINE OSTEOCYTES ONTO A MICROFLUIDIC PLATFORM FOR OBSERVING BONE METASTASIS

This protocol describes the integration of primary osteocytes onto a microfluidic platform developed by our group to observe cancer cell extravasation towards bone cells (Seaman et al., 2025). Herein, we describe the establishment of a hydrogel lumen and the seeding of cells for microfluidic co‐culture (Mei et al., 2019). Following this protocol allows for the microfluidic culture of primary osteocytes for 5 days and the observation of cancer cell extravasation towards primary osteocytes. Given that microfluidic platforms all have distinct geometries and design, we outline and annotate key considerations that were made to produce the results with primary osteocytes shown in Seaman et al. (2025). We describe this protocol using the same cancer cell and endothelial cell lines used in Seaman et al. (2025) However, we would like to note that other cancer or endothelial cell lines can be cultured in this device as long as they are cultured in their associated growth medium.

### Materials

Sylgard 184 silicone elastomer kit (Dow, 4019862)
70% ethanol
DPBS (Sigma‐Aldrich, D8537‐500ML)
0.15 mg/ml collagen solution (see recipe)
Matrigel, growth factor reduced (Corning, 356231)
Fibronectin (Sigma‐Aldrich, F1141‐2MG)
5× DPBS (diluted from 10×; Sigma, D1408‐500ML)
5.0 M NaOH (VWR, DH72471)
High concentration collagen type I (Corning, 354249)
Human umbilical vein endothelial cell (HUVEC) growth medium (see recipe)
Primary osteocyte medium (see recipe)
HUVECs (gift from Dr. Craig Simmons, University of Toronto)
CellTracker Orange CMTMR (Invitrogen, C2927)
Primary osteocytes (see Basic Protocol 2)
PC‐3 prostate cancer cells (ATCC)
CellTracker Green CMFDA (Invitrogen, C2925)
PC‐3 growth medium (see recipe)

Oven
Glass slides (Corning, 2947‐75 × 50)
Plasma cleaner
Hot plate
Biosafety cabinet (Thermo Scientific, Model 1355 Class A2)
Square dishes, sterile (Fisherbrand, 0875711A‐10PK)
Water bath (empty pipette box with distilled water)
Container with ice
Vortex mixer (Fisher Scientific, Standard 120V)
Aspirator (Gast, 0523‐101Q‐G180DX)
Cell culture incubator, 37°C and 5% CO_2_ (Thermo Scientific Forma Steri‐Cycle CO_2_ Incubator, 184 L)
Tissue culture microscope (Zeiss, Z‐AXIO40C)
Fluorescent microscope for imaging (Nikon Eclipse Ti‐U inverted microscope)

1 Create polydimethylsiloxane (PDMS) devices from an SU‐8 master by mixing the base and curing agent from the Sylgard 184 silicone elastomer kit at a 10:1 ratio and baking at 55°C overnight.
2 Bond devices to clean glass slides by plasma (O_2_) treatment and baking on a hot plate at 80°C for 3 min.
3 In a biosafety cabinet, sterilize the exterior of the devices with 70% ethanol and tape the microfluidic devices to square Petri dishes.
4 Sterilize microfluidic channels by rinsing in 70% ethanol three times. Rinse the sterilized channels five times with DPBS.
5 Coat the channel for osteocytes in 0.15 mg/ml collagen I for 1 hr at room temperature. 100 µl of collagen solution is sufficient for one device containing six channels. Ensure that the collagen only coats the osteocyte channel and that there is no excess at the outlets.
6 During collagen coating, thaw Matrigel (growth factor reduced) at 4°C and prepare a water bath using an empty pipette box containing distilled water.
7 Aspirate all collagen from the osteocyte channels.
8 Coat both osteocyte and lumen channels with a 100 µg/ml fibronectin solution to promote adhesion of the hydrogel to the channel. Incubate the devices at 4°C for 40 min in a sterile container.
9 Prepare the hydrogel mixture for the lumen channel.
  a. Start by dispensing 50 µl of 5× DPBS and 0.78 µl of 5.0 M NaOH and mixing.
  b. Using an ice‐cold pipette tip, add 144.8 µl high concentration collagen I (11.07 mg/ml), swirling the solution gently to ensure a uniform mixture.
  c. Promptly mix the hydrogel with a vortex mixer and incubate for 5 min on ice.
  d. Add 95.8 µl of Matrigel (7.6 mg/ml) into the hydrogel and mix using a vortex mixer again.
  e. The final concentrations of collagen I and Matrigel are 5.5 mg/ml and 2.5 mg/ml, respectively.
    If new stocks of collagen I and Matrigel are purchased, the required volumes of collagen I and Matrigel components need to be recalculated.
10 Aspirate the fibronectin out of al channels just prior to adding the hydrogel.
11 With an ice‐cold pipette tip, slowly add 10 µl of the hydrogel into the lumen channel.
12 Remove the hydrogel after 30 s, ensuring the bulk of the hydrogel has been removed.
  Having excessive residual hydrogel can clog the channel. To form a 3D lumen, the hydrogel only needs to coat the perimeter of the channel.
13 Ensure that the side channels are filled with hydrogel. If needed, carefully aspirate from the inlet of the osteocyte channel to fill the side channels with hydrogel.
14 Incubate the device inside the water bath at 37°C for 45 min to polymerize the hydrogel.
15 Add warm HUVEC medium to the lumen channel, and warm DPBS to the osteocyte channel.
16 Ensure the channels are not mixing, as this means the hydrogel is broken.
17 Remove the DPBS from the osteocyte channel and replace with fresh primary osteocyte medium.
  Ensure that liquid flows properly through all the channels.
18 Place devices in cell culture incubator overnight for cell seeding the next day.
19 Stain HUVECs with CellTracker Orange. Prepare a HUVEC suspension at a density of 2 × 10^6^ cells/ml.
20 Remove excess medium from the outlet and add 10 µl of the HUVEC suspension while tilting the device to the left. Incubate the devices at 37°C, 5% CO_2_ for 20 min while still tilted to the left.
21 Remove excess medium from the outlet of the lumen channel and seed 10 µl of the HUVEC suspension again in an upright position. Incubate for 20 min at 37°C, 5% CO_2_.
22 Repeat step 20 twice more, tilting right and upside down to create an endothelial lumen. Confirm that endothelial cells are adhered to the walls of the lumen under a tissue culture microscope.
23 Allow endothelial cells to form a network, do not disturb the lumen in the devices for at least 6 hr.
24 Prepare a suspension of primary osteocytes at a density of 1.25 × 10^6^ cells/ml, following Basic Protocol 2.
  We seed fewer primary osteocytes compared to MLO‐Y4 cells (which are seeded at 1.5 × 10^6^ cells/ml). This is to ensure that the primary osteocytes did not become too confluent as they are larger than MLO‐Y4 cells. We were also unaware of whether primary osteocytes undergo contact inhibition in vitro, we have not confirmed this effect. Moreover, we wanted to ensure that primary osteocytes would have access to sufficient nutrients/supplements in the medium, as microfluidic environments in general limit the availability of nutrients to cells.
25 Remove half of the medium from the outlet of the osteocyte channel. Add 15 µl of the primary osteocyte cell suspension to the osteocyte channel.
26 Incubate primary osteocytes for at least 5 hr prior to seeding cancer cells in the lumen channel.
  We have noted that primary osteocytes take longer to spread than MLO‐Y4 cells, which require 3 hr of undisturbed culture (compared to 5 hr) prior to seeding cancer cells. The primary osteocytes should still spread out in the channel and exhibit a dendritic morphology. Depending on the device geometry and channel volume, the time that primary cells take to spread in the channel should be noted.
27 Stain PC‐3 cancer cells with CellTracker Green. Prepare a suspension of 4 × 10^6^ cells/ml.
28 Gently add 18 µl of the PC‐3 cancer cell suspension to the lumen channel. Incubate the microfluidic cultures for 20 min at 37°C, 5% CO_2_.
29 Gently add 20 µl of the cancer cell suspension to the lumen channel while tilting to the left. Incubate again for 20 min at 37°C, 5% CO_2_.
30 Add PC‐3 growth medium to the outlet and culture overnight.
31 Maintain microfluidic cultures for at least 4 days to observe cancer cell extravasation, changing the medium in each channel twice daily (at least 8 hr apart). The lumen channel is maintained with a 1:1 mixture of cancer cell and endothelial cell medium, whereas primary osteocytes are maintained in their culture medium (primary osteocyte medium). Image extravasating cancer cells under a fluorescent microscope throughout the experiment.
  We have found that while MLO‐Y4 cells can be maintained with daily medium changes, primary osteocytes begin shrinking. For other experimental setups, the frequency of medium changes for primary cells may need to be optimized.

## REAGENTS AND SOLUTIONS

*All reagents and solutions are to be prepared in sterile conditions*.

### Alexa Fluor 488 secondary antibody solution

400 µl of 7.5% (w/v) BSA solution (1% w/v final; see recipe)
30 µl Alexa Fluor 488 donkey anti‐goat IgG antibody (1:100 antibody dilution by volume; Invitrogen, A11055)
2.56 ml DPBS (Sigma‐Aldrich, D8537‐500ML)
Prepare and use fresh

### α‐MEM ± 10% P/S sterilizing solution

45 ml α‐MEM (Gibco, 12571063)
5 ml penicillin‐streptomycin (P/S) (10% final; Gibco, 15140122)
Prepare the day before primary cell extraction and store at 4°C

### Blocking buffer (immunofluorescence)

400 µl of 7.5% (w/v) BSA solution (1% final; see recipe)
150 µl normal donkey serum (5% v/v final; Sigma‐Aldrich, D9663‐10ML)
2.55 ml DPBS (Sigma‐Aldrich, D8537‐500ML)
Prepare and use fresh

### Bovine serum albumin (BSA) solution, 7.5%

7.5 g BSA (BioShop, ALB005.50)
100 ml sterile DPBS (Sigma‐Aldrich, D8537‐500ML)
Store up to 3 months at 4°C

### Collagen‐coated plates

Dispense 4 ml of 0.15 mg/ml collagen solution (see recipe) onto 100‐mm cell culture dishes (Corning, 430167) using a chilled pipette tip. Coat dishes for 1 hr at room temperature. Remove the collagen solution and let dishes dry. Store dishes up to 1 month at 4°C.

### Collagen solution, 0.15 mg/ml

4.40 ml rat tail collagen type I (0.15 mg/ml final; Corning, 354236)
95.6 ml of 0.02 M glacial acetic acid (Sigma‐Aldrich, A6283‐500ML)
Add collagen to acid
Store up to 3 months at 4°C
  Collagen solution can be used ≤5 times.

### DPBS/BSA solution for transport

50 ml Dulbecco's phosphate‐buffered saline (DPBS) (Sigma‐Aldrich, D8537‐500ML)
0.5 g BSA (1% w/v final; BioShop, ALB005.50)
Prepare the day before primary cell extraction and store at 4°C

### E11/podoplanin primary antibody solution

400 µl of 7.5% (w/v) BSA solution (1% w/v final; see recipe)
30 µl mouse E11/podoplanin antibody (1:100 antibody dilution by volume; R&D Systems, AF3244)
2.56 ml DPBS (Sigma‐Aldrich, D8537‐500ML)
Prepare and use fresh

### EDTA solution

49 ml DPBS (Sigma‐Aldrich, D8537‐500ML)
0.5 ml of 0.5 M ethylenediaminetetraacetic acid (EDTA) solution in DPBS (5 mM final; Sigma‐Aldrich, E6511‐100G)
0.67 ml of 7.5% BSA solution (0.1% w/v final; see recipe)
Prepare the day before primary cell extraction and store at 4°C

### HUVEC growth medium

500 ml Endo Max medium (87% v/v final; Wisent, 301‐010 CL)
56 ml fetal bovine serum (FBS) (10% v/v final; Gibco, 12483020)
11.5 ml Endo Max endothelial cell growth supplement (2% v/v final; Wisent, 301‐012 XL)
5.75 ml P/S (1% v/v final; Gibco, 15140122)
Store up to 3 months at 4°C

### Liberase TM solution, 13 Wünsch U/ml

5 mg Liberase TM, research grade, 1 × 5 mg vial (Roche, 5401119001)
2 ml dH_2_O, sterile
Prepare the day of primary cell extraction and store at 4°C in between digestions
Store remaining reconstituted solution in single‐use aliquots up to 1 month at –20°C

### PC‐3 growth medium

500 ml F‐12K medium (Kaighn's modification) (89% v/v final; Gibco, 21127022)
56.2 ml FBS (10% v/v final; Gibco, 12483020)
5.6 ml P/S (1% v/v final; Gibco, 15140122)
Store up to 3 months at 4°C

### Primary osteocyte medium

500 ml α‐MEM (89% v/v final; Gibco, 12571063)
28.1 ml FBS (5% v/v final; Gibco, 12483020)
28.1 ml newborn calf serum (5% v/v final; Gibco, 16010159)
5.6 ml P/S (1% v/v final; Gibco, 15140122)
Prepare the day before primary cell extraction and store at 4°C

### Working medium for imaging primary osteocytes

49 ml α‐MEM (98% v/v final; Gibco, 12571063)
0.5 ml FBS (1% v/v final; Gibco, 12483020)
0.5 ml P/S (1% v/v final; Gibco, 15140122)
Prepare the day before calcium imaging and store at 4°C

## COMMENTARY

### Critical Parameters

The successful extraction of osteocytes from murine long bones depends on the time taken to process samples prior to digestion. We highly recommend planning the workflow for processing samples beforehand to reduce the time spent on these steps to begin enzyme and EDTA digestions as quickly as possible. After cells have been extracted, it is important to ensure that cells attach to the plate over the subsequent period of culture to enhance the yield of cells. Because residues from extraction reagents can be harmful to cells, we recommend performing partial medium changes to dilute the concentration of these reagents. A complete medium change can be performed once cells have attached.

We do not recommend culturing primary cells for >2 weeks, as primary cells can lose their phenotype over longer periods of time. Regarding the subculture of primary osteocytes, the trypsinization step is key to acquiring a good yield of primary cells for seeding experiments; we have observed the primary cells can be difficult to detach from their culture plate. Therefore, primary cell cultures should be incubated with enough trypsin and not be disturbed during incubation to enhance the yield of detached cells. We also perform trypan blue staining to ensure that cells are still viable during passaging.

Critical elements for the calcium imaging of primary osteocytes include proper rinsing of medium or dye components during the staining of samples and the setup for applying oscillatory fluid flow. Specifically, primary osteocyte medium must be completely rinsed from the samples prior to staining as serum components can interfere with FURA‐2 AM dye during sample incubation. Moreover, samples must be thoroughly rinsed after staining to mitigate excessive background signals, as residual dye molecules will interfere with ratiometric calcium measurements. Bubbles cannot be present in the tubing or connections for applying fluid flow under any circumstances, as this can affect the peak shear stress applied to samples. Bubble present in the channel during the application of fluid flow kills cells, thereby ending the experiment.

Important considerations for the incorporation of primary osteocytes onto microengineered platforms include the initial seeding density and frequency of medium changes (or access to nutrients) in microfluidic channel. Here, we describe the protocol to seed and culture primary osteocytes on the microfluidic platform developed by our research group used in Seaman et al. (2025) to observe the extravasation of prostate cancer to bone. Consideration of these factors can change with the use of other platforms depending on the cell growth area, channel volume, device geometry, and experimental setup (e.g., static or perfusion culture).

### Troubleshooting

Commonly observed problems, their possible causes and suggestions to address the issue are listed below in Table 1.

**Table 1:** Troubleshooting Guide for the Extraction and Use of Primary Osteocyte for In Vitro Studies

Problem	Possible Cause	Solution
Low cell yield during digestions	Liberase TM enzyme or EDTA activity affected	Perform extra washes in between fractions
Cells not attaching to collagen‐coated plate in days following extraction	Extraction residues reducing cell viability	Add 2‐3 ml extra medium to each dish; do not remove any medium during first 72 hr after extraction; can later perform partial medium changes so as not to remove too many cells
Cells not detaching from plate during trypsinization	Insufficient quantity of trypsin, or incubation was disturbed	Incubate samples for an extra 1‐2 min at 37°C or add 1 ml extra trypsin (for a 10‐cm plate); gently tap the sides of the dish to help detach cells
Primary cells appear rounded after FURA‐2 AM staining	Samples incubated too long at room temperature	Reduce the time spent staining samples at room temperature in 5‐min increments
Excessive background or static noise during calcium imaging	FURA‐2 AM dye was not rinsed properly from the channels	Add an extra rinse of DPBS during the washing step
No FURA‐2 signal	FURA‐2 AM dye was not mixed properly before staining or interference from serum components in culture medium	For every sample, vortex mix the FURA‐2 staining solution well before staining; add an extra rinse of DPBS before staining samples with FURA‐2 AM
Primary cells do not produce a ratiometric calcium response	Imaging was not performed near the middle of the channel	Ensure that the area being imaged is not near the channel wall and is instead in the middle of the channel; include a control or reference group to confirm the calcium response
Primary cells not attaching or spreading out in microfluidic channel	Insufficient collagen I/extracellular matrix (ECM) to promote attachment or cell spreading	While the collagen coating solution can be reused up to five times, use a freshly prepared collagen solution to coat the microfluidic channels; addition of other ECM molecules such as fibronectin would help promote cell attachment and spreading
Primary cells detach from microfluidic channel over time	Cells do not have sufficient access to nutrients	Increase the frequency of medium changes or provide perfusion to replenish medium

### Understanding Results

**Figure 2:**
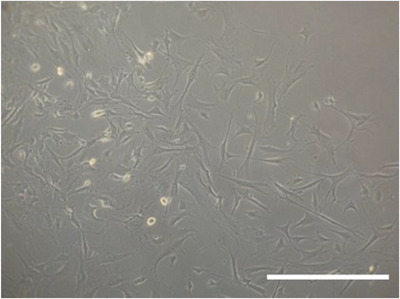
Primary osteocytes post‐extraction. Scale bar = 100 µm.

**Figure 3:**
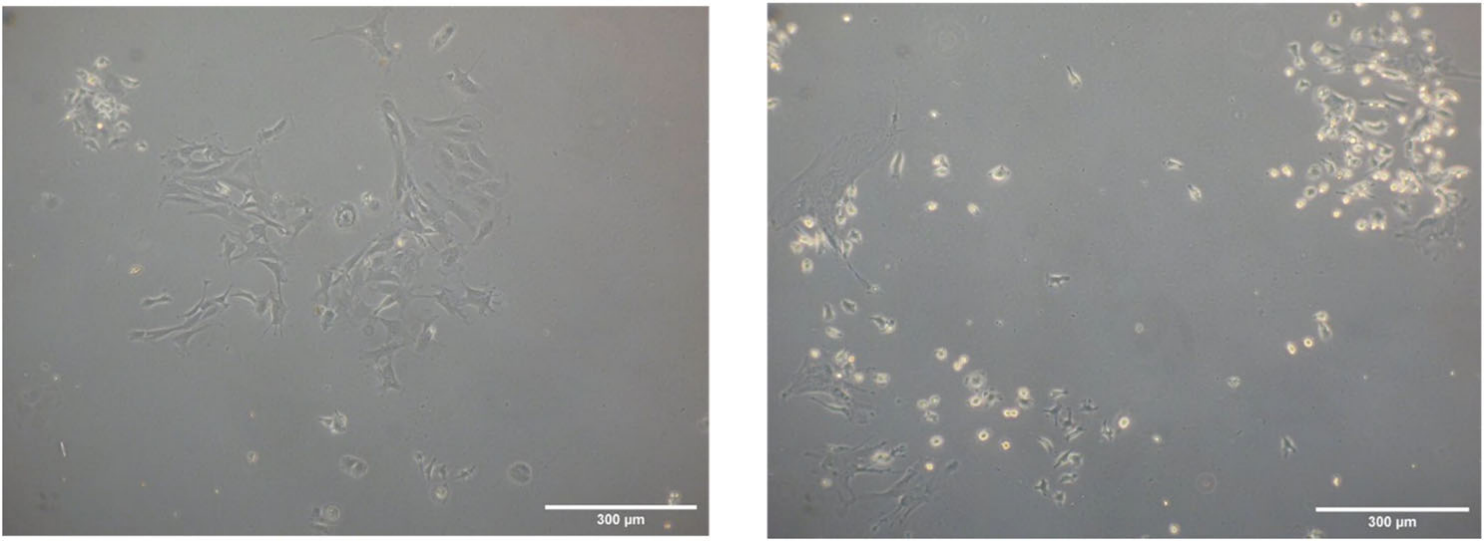
Primary osteocytes 4‐days‐post extraction using Liberase TM or collagenase enzymes.

**Figure 4:**
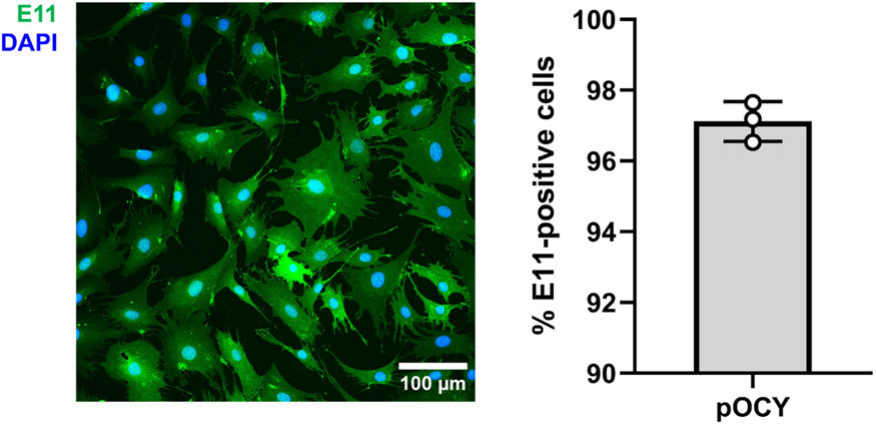
E11 staining of primary osteocytes extracted from 2‐month‐old male mice, with the percentage of E11 positive cells from *n* = 3 samples.

In this protocol we used Liberase TM, a high purity blend of collagenases, rather than type I collagenase to digest collagen in bone. This was mainly attributed to observed differences in cell cultures post‐extraction when type I collagenase vs Liberase TM was used. We have noted that primary cultures extracted with Liberase TM tend to have more cells spread out in the culture dish. More importantly, cells exhibit dendritic morphology typical of osteocytes as observed in Figures 2 and 3. We therefore use LiberaseTM to extract primary osteocytes in our laboratory. While type I collagenase has been used the literature for the extraction of primary osteocytes, we anticipate this article presents an alternative approach to isolate primary osteocytes from murine long bones (Gooi et al., 2019; Shah et al., 2016; Stern et al., 2012). Primary osteocytes should attach to the culture dish 1 week after extraction. The yield of primary cells in a 100‐mm culture dish after extraction typically ranges from 1 to 2 million cells, depending on several factors, such as the age of mice and the number of long bones pooled together during extraction. Based on E11/podoplanin immunofluorescence staining results shown in Figure 4, the extraction of primary osteocytes should yield a relatively high osteocyte population (95% to 97%).

**Figure 5:**
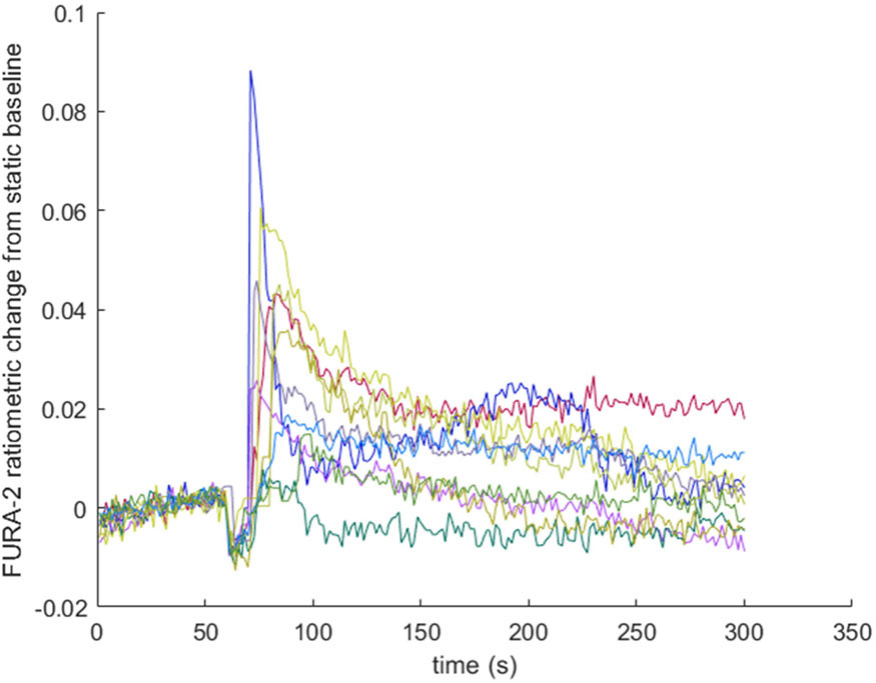
Recorded ratiometric calcium data from 10 primary osteocytes over the course of 5 min. The static baseline was recorded during the first 60 s of the experiment.

**Figure 6:**
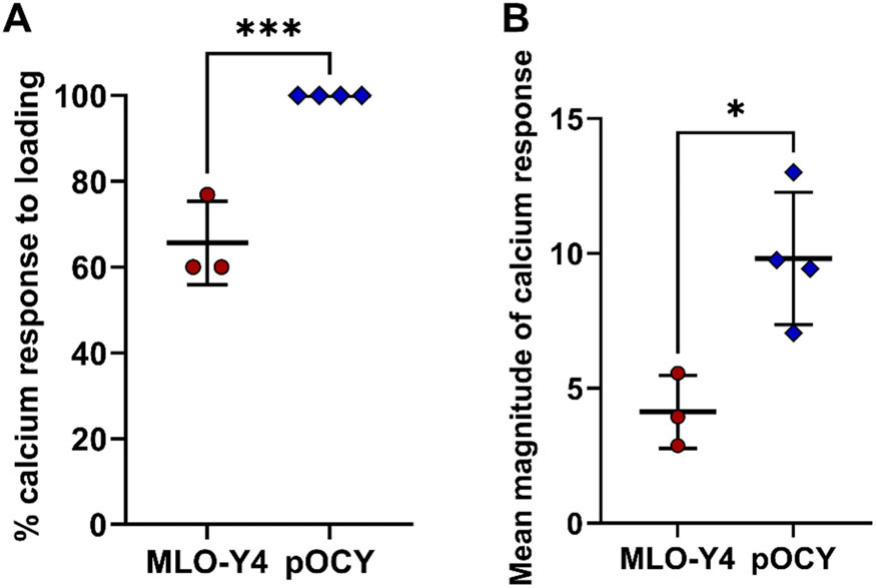
Data obtained from calcium imaging of primary osteocytes (pOCY) from 2‐month‐old female mice. The MLO‐Y4 osteocyte‐like cell was used as a control for reference. (**A**) Percentage of cells with a significant response to oscillatory fluid flow. ***p < .001 (two‐tailed *t*‐test) (**B**) Mean magnitude of the recorded calcium response. *p < .05 (two‐tailed *t*‐test).

Following extraction, we performed real‐time calcium imaging to determine whether the primary osteocytes (2‐month‐old female mice) could produce a functional response upon exposure to physiological levels of fluid shear stress (mechanical loading). We used FURA‐2 AM and EasyRatio Pro imaging software to record 340/380 nm ratiometric data from cells in an area of interest. Here, excitation of FURA‐2 at 340 nm represents excitation of bound FURA‐2/Ca complexes, whereas 380 nm is the excitation wavelength for unbound FURA‐2 molecules. During osteocyte mechanotransduction, fluid shear stress induces Ca influx through ion channels, such as Piezo1 or TRPV4 (Qin et al., 2020). Figure 5 therefore shows the change in the ratio between bound to unbound FURA‐2 molecules compared to the static baseline with no fluid flow. The ratiometric change from the static baseline response of each cell on the image display was captured in the recording for the entire duration of the experiment, resulting in the observation of ≥10 osteocytes per sample. The recorded raw data of the FURA‐2 ratiometric change for *n* = 10 primary osteocytes is presented in Figure 5. Figure 6 presents calcium response data from the MLO‐Y4 cell line and primary osteocytes from 2‐month‐old female mice. We used the MLO‐Y4 cell line as a reference group to compare with primary osteocytes. Here, we measured the percentage of cells with a significant Ca influx in response to mechanical loading as well as the mean magnitude of the calcium response. For these data, the calcium response of osteocytes was only considered significant if the magnitude of the response was twice as high as the static baseline that was measured before fluid shear stress was applied.

**Figure 7:**
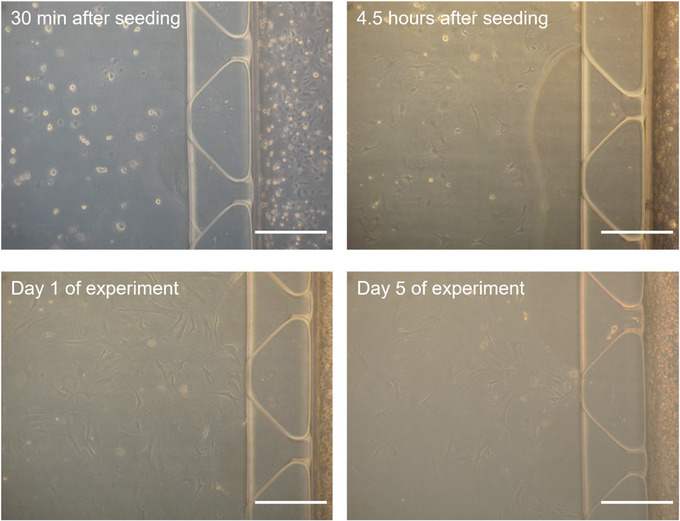
Incorporating primary osteocytes into a microfluidic platform to study early‐stage prostate cancer bone metastasis (extravasation). Brightfield images taken at various timepoints from seeding primary cells to the final day of experiment (note: images taken in low light settings). Scale bars = 300 µm.

Figure 7 shows the seeding and maintenance of primary osteocytes in a microfluidic co‐culture platform. Primary osteocytes could indeed be maintained in co‐culture for 5 days. In the study by Seaman et al. (2025), primary osteocytes from 2‐month‐old male mice were incorporated into this system to demonstrate that mechanical loading of osteocytes under physiological levels of oscillatory fluid flow during exercise (1 Pa, 1 Hz, 2 hr) could regulate prostate cancer cell extravasation through a microfluidic lumen lined with endothelial cells. These results aligned well with experiments performed using the MLO‐Y4 osteocyte cell line (Seaman et al., 2025). Since primary osteocytes can be successfully extracted and validated for a functional mechanoresponse using this protocol, we anticipate that these cells can be used to enhance the biological relevance of in vitro osteocyte mechanobiology studies.

### Time Considerations

Basic Protocol 1 can be performed in 10 to 12 hr, depending on the number of individuals working to extract primary cells and general familiarity with the protocol. Basic Protocol 2 describing the subculture of primary cells takes 30 min; E11/podoplanin staining can be performed in 24 hr. Samples are fixed, permeabilized and incubated in blocking buffer over 2.5 to 3 hr prior to incubation overnight with the primary antibody. Incubation with the secondary antibody and DAPI counterstaining takes 3 hr the following day. Basic Protocol 3 on real‐time calcium imaging of primary osteocytes can be completed in 24 hr. Cell seeding of samples prior to overnight culture and calcium imaging takes 3 to 3.5 hr (depending on the number of samples). Real‐time calcium imaging can take 5 to 6 hr to complete the next day depending on the number of samples. The experiment in Basic Protocol 4 takes 6 days to complete. The hydrogels take 4 to 5 hr to complete based on the number of devices used. Cell seeding takes 8 to 9 hr to complete the next day. Cells were cultured in microfluidic devices for another 4 to 5 days.

### Author Contributions

**Kimberly Seaman**: Conceptualization; data curation; formal analysis; investigation; methodology; writing—original draft. **Chun‐Yu Lin**: Data curation; investigation; methodology. **Xin Song**: Data curation; methodology. **Amel Sassi**: Investigation; methodology. **Weidong Du**: Methodology. **Yu Sun**: Supervision. **Burton Yang**: Resources; supervision. **Lidan You**: Conceptualization; formal analysis; funding acquisition; investigation; project administration; resources; supervision; writing—review and editing.

### Conflict of Interest

The authors declare no conflict of interest.

## Data Availability

The data, tools, and material (or their source) that support the protocol are available from the corresponding author upon reasonable request.
